# Genomic landscape of the immune microenvironments of brain metastases in breast cancer

**DOI:** 10.1186/s12967-020-02503-9

**Published:** 2020-08-31

**Authors:** Wei-cheng Lu, Hui Xie, Ce Yuan, Jin-jiang Li, Zhao-yang Li, An-hua Wu

**Affiliations:** 1 Department of Neurosurgery, First Affiliated Hospital of China Medical University, Shenyang, Liaoning China; 2 Department of Histology and Embryology, College of Basic Medicine, Shenyang Medical College, Shenyang, Liaoning China; 3 Graduate Program in Bioinformatics and Computational Biology, University of Minnesota, Minneapolis, USA; 4 Department of Neurosurgery, General Hospital of Northern Theater Command, Shenyang, Liaoning China; 5 Department of Laboratory Animal Center, China Medical University, Shenyang, Liaoning China

**Keywords:** Breast cancer, Brain metastases, Immune, Gene

## Abstract

**Background:**

This study was intended to investigate the genomic landscape of the immune microenvironments of brain metastases in breast cancer.

**Methods:**

Three gene expression profile datasets (GSE76714, GSE125989 and GSE43837) of breast cancer with brain metastases were downloaded from Gene Expression Omnibus (GEO) database. After differential expression analysis, the tumor immune microenvironment and immune cell infiltration were analyzed. Then immune-related genes were identified, followed by function analysis, transcription factor (TF)-miRNA–mRNA co-regulatory network analysis, and survival analysis of metastatic recurrence.

**Results:**

The present results showed that the tumor immune microenvironment in brain metastases was immunosuppressed compared with primary caner. Compared with primary cancer samples, the infiltration ratio of plasma cells in brain metastases samples was significantly higher, while the infiltration ratio of macrophages M2 cells in brain metastases samples was significantly lower. Total 42 immune-related genes were identified, such as *THY1* and *NEU2*. *CD1B*, *THY1* and *DOCK2* were found to be implicated in the metastatic recurrence of breast cancer.

**Conclusions:**

Targeting macrophages or plasma cells may be new strategies for immunotherapy of breast cancer with brain metastases. *THY1* and *NEU2* may be potential therapeutic targets for breast cancer with brain metastases, and *THY1*, *CD1B* and *DOCK2* may serve as potential prognostic markers for improvement of brain metastases survival.

## Background

The incidence of brain metastases in cancer patients is rising, which may be due to the improvements in systematic therapies to control extracranial disease and prolong the survive of patients. Thus, patients who previously may have died sooner from other manifestations of the disease may develop brain metastases [1]. Breast cancer is the second most common cause of brain metastases following lung cancer [2]. It has been estimated that 20 to 30% of breast cancers develop brain metastases [3]. Brain metastases are serious complications of cancer with median survival of about 15 months and there is no effective long-term treatment [4, 5]. Therefore, brain metastases have become a major limiting factor in life expectancy and quality of life for many patients [2]. Understanding the biological mechanisms of brain metastases is crucial to predict patients at risk of brain metastases and to identify new therapeutic targets.

The interactions between immune and tumor cells have played an important role in malignant progression [6]. The brain was previously considered as an immunologically privileged organ because the intact brain has almost no lymphocytes [7]. Actually, the central nervous system is an immune specialized site under a tight regulatory network linking astrocytes, microglia, and lymphocytes [8]. T cells and B cells have been found around the tumors of brain metastases [9]. Despite the immune microenvironments of brain metastases in breast cancer have been studied [10, 11], the genomic landscape of breast cancer with brain metastases remains to be investigated.

**Fig. 1:**
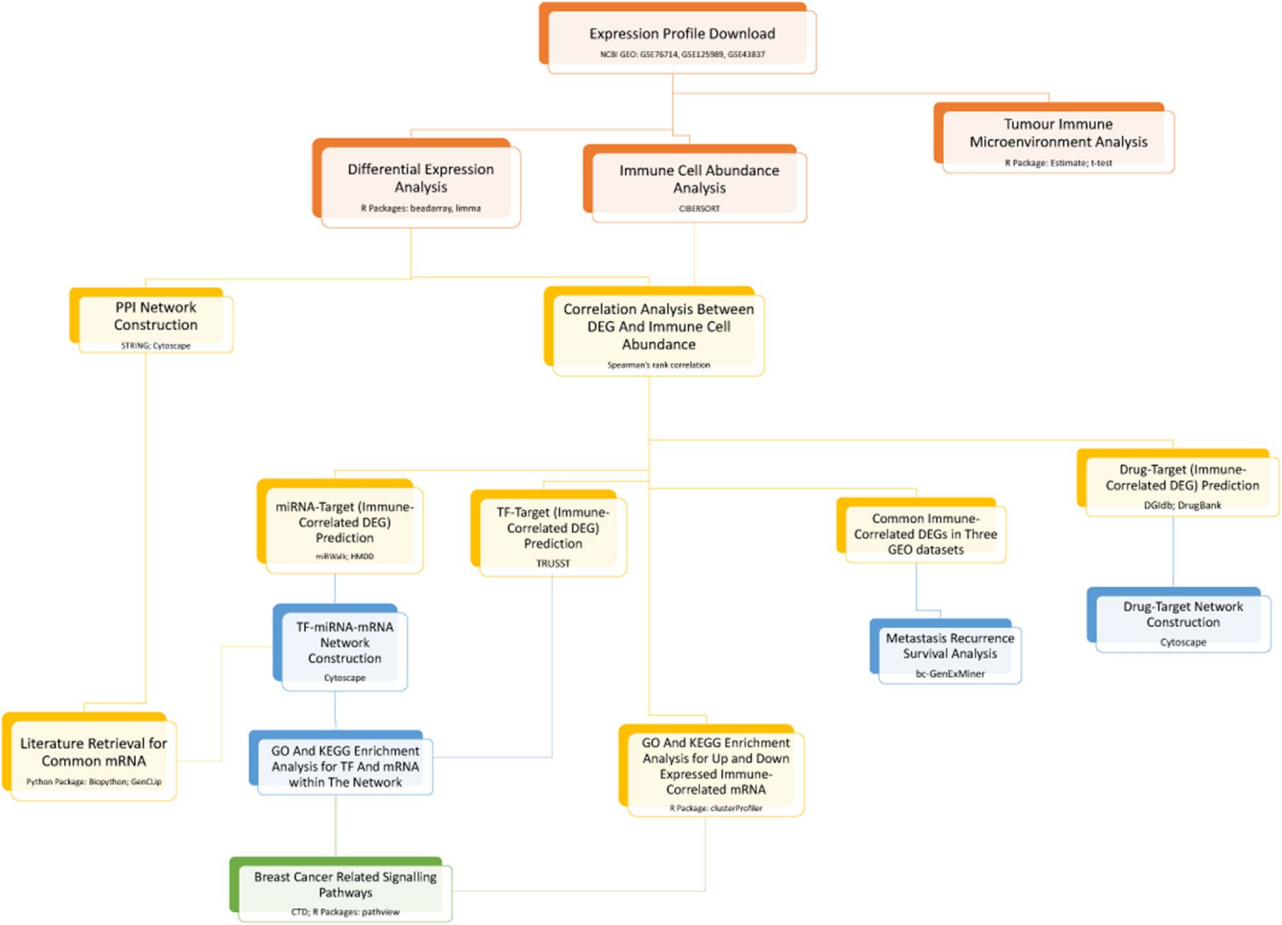
The flow diagram of the analysis

In this study, we downloaded three gene expression profile datasets of breast cancer with brain metastases from Gene Expression Omnibus (GEO) database and analyzed the tumor immune microenvironment at genetic level (Fig. 1). Some immune-related genes were identified, which may contribute to the development of immunotherapy to treat breast cancer patients with brain metastases.

## Methods

### Data sources

Three expression profile datasets (GSE76714 [12], GSE125989 [1] and GSE43837 [13]) were downloaded from GEO database. GSE76714 included 71 triple negative breast cancer samples, including 48 primary triple negative breast cancer samples and 23 triple negative breast cancer with brain metastases samples, which was based on the platform of GPL14951 Illumina HumanHT-12 WG-DASL V4.0 R2 expression beadchip. GSE125989 contained 16 primary breast cancer samples, 16 paired breast cancer with brain metastases samples. The detection platform was GPL571 [HG-U133A_2] Affymetrix Human Genome U133A 2.0 Array. In GSE43837, there were 19 HER2 + primary breast cancer samples and 19 HER2 + breast cancer with brain metastatic samples. The platform was GPL1352 [U133_X3P] Affymetrix Human X3P Array.

### Data downloading and preprocessing

The series matrix file(s) of GSE76714 was downloaded and normalized using quantile normalization with the beadarray library in R. The probe ID was converted into gene symbol using illuminaHumanWGDASLv4 package in R. The probes that did not correspond to gene symbol were removed. For the case where different probes mapped to the same gene, the mean value of different probes was taken as the final expression value of the gene. The FactoMineR package [14] was used for principal component analysis and clustering. The processing flows of GSE125989 and GSE43837 datasets were similar to that of GSE76714. GSE43837 data were transformed by log2(x + 1), and GSE125989 data were normalized by MAS5 algorithm of R language package, and transformed by log2. The microarray annotation packages of the GSE125989 and GSE43837 datasets were u133x3p.db and hgu133a2.db, respectively.

### DEGs identification

The modified empirical Bayes *t* test method provided by limma package [15] (version 3.40.6) was used for differential expression analysis of brain metastasis group vs. cancer group. All RNAs (including mRNAs and lncRNAs) were analyzed to obtain the p value and log fold change (FC). The thresholds of DEGs screening were set as follows: p value < 0.05 and |logFC| > 0.585.

The ggscatter function of ggpubr package [16] (version 0.2.2) was used to draw the volcano plot, and the DEGs with the top 10 |logFC| were labeled in the volcano plot. The clustering heatmaps of DEGs were drawn using the pheatmap package [17] of R language.

### Tumor immune microenvironment analysis

The stromal score, immune score and ESTIMATE score of all samples were calculated using the ESTIMATE algorithm [18] (version 1.0.13). The difference of these scores between brain metastatic tumor and primary tumor tissues were analyzed through T test, and the boxplot was drawn by using the R package ggpubr. Additionally, the cytolytic activity score of all samples was calculated and the differences of score between brain metastatic tumor and primary tumor tissues were also analyzed using T test. The boxplot was drawn by the R package ggpubr as well. Validation for the above scores was performed in the GSE125989 and GSE43837 datasets.

### Immune cell infiltration abundance analysis

The abundance matrix of immune cells in the samples was estimated using the CIBERSORT deconvolution algorithm [19], and the infiltration abundance of immune cells in the samples was analyzed, with parameters of perm = 200 and QN = FALSE. The proportion difference of immune cell subgroups between two groups was calculated, and relevant landscape map (barplot), clustering heatmap (pheatmap), correlation heatmap (corHeatmap), and violin plot (vioplot) were drawn by R language. The immune cell subgroups with significant differences between groups were screened with threshold of p value < 0.05. The datasets of GSE125989 and GSE43837 were used for validation.

### Identification of immune-related DEGs

Using the R corrplot package [20], spearman correlation test was conducted on the DEGs and infiltration abundance of differential immune cell subsets, and the DEGs with p value < 0.05 and correlation coefficient |r| > 0.30 were screened, which were considered as the DEGs related to immune cell subgroup. The ggboxplot function of the ggpubr package in R language was used to plot the boxplot of the expression of immune-related genes between two groups, and the differences of these genes between the two groups were further analyzed by T test. The datasets of GSE125989 and GSE43837 were used for validation.

### Function and pathway enrichment analyses

These immune-related DEGs were subjected to Gene Ontology (GO) [21] and KEGG [22] pathway using ClusterProfiler [23] (version 3.12.0). The GO analysis results included biological process (BP), cellular component (CC) and molecular function (MF). The significance threshold was p value < 0.05, and the enrichment number (count) was at least 2. The compareCluster function of the clusterProfiler package was applied to visualize the top 10 GO BP and KEGG enrichment results.

### Transcription factor (TF)-miRNA-mRNA co-regulatory network analysis

The miRNAs in the 3′UTR region of immune-related DEGs were predicted using relevant databases (miRWalk3.0 [24], TargetScan [25], miRDB [26], mirTarBase [27]), with a threshold score of > 0.95. Combining the results from each database, miRNAs that were validated (MirTarBase) and predicted in at least one other database were selected as the final mRNA-miRNA relationship pairs. HMDD V3.2 database [28] was used to retrieve the keyword “breast neoplasms” (synonyms of breast cancer) to further validate the predicted miRNAs. Then based on the online database TRRUST [29], the TF-mRNA pairs associated with immune-related DEGs and possible action mode (activation, suppression or unknown) were predicted. The mRNA-miRNA relationship pairs and TF-mRNA relationship pairs were integrated to construct the network using Cytoscape [30]. Additionally, GO and KEGG analyses were performed for the TF and immune-related DEGs in the network.

### Breast cancer associated pathway screening and gene annotation

According to the breast neoplasms related pathways included in Comparative Toxicogenomics Database (CTD) [31], further screening was conducted for the KEGG pathways enriched by immune-related genes as well as transcription factors and immune-related genes in the network. The R package pathview [32] (version 1.24.0) was applied to draw the pathway map.

### Protein-protein interaction (PPI) network analysis

The interaction between gene coding proteins was predicted and analyzed using STRING database [33] (version 11.0). The input gene set was immune-related genes, and the PPI score was set as 0.4 (medium confidence). After the PPI relation pairs were obtained, Cytoscape software was used to construct a network. Network topology properties (betweenness, closeness and degree) were analyzed using CytoNCA [34] plug-in of Cytoscape software. Functional modules in the network were identified using MCODE [34] plug-in of Cytoscape. Parameters were set as default, and the sub-network modules were screened according to score ≥ 4.

### Drug-gene interaction prediction

DGIdb 3.0 [35] (version 3.0.2) database was applied to predict the drug-gene interaction of immune-related genes, and the parameters were set as default. The drug-gene interaction network was constructed through Cytoscape. The predictive drug information was retrieved in the DrugBank [36] database.

### Metastatic recurrence survival analysis of key genes

The bc-GenExMiner v4.4 [37] online tool was used for breast cancer gene expression data mining, and the immune-related genes that were verified by data were subjected to metastatic recurrence survival analysis. The parameters were as follows: baseline like (PAM50) and/or triple-negative breast cancer (IHC) prognostic analysis; DNA microarrays samples (n = 10,001); metastatic recurrence; segmentation criteria of median.

### Literature retrieval of key genes

The genes in PPI network and TF-miRNA–mRNA network were considered as key genes. The Biopython Python package [38] was used to access the NCBI Entrez database, and GenCLiP 2.0 database [39] was used to summarize the breast cancer associated literatures related to these key genes. The title and abstract were retrieved, and the literatures were considered as relevant literatures if both gene and disease keyword appeared.

## Results

### Data preprocessing and DEGs analysis

**Fig. 2:**
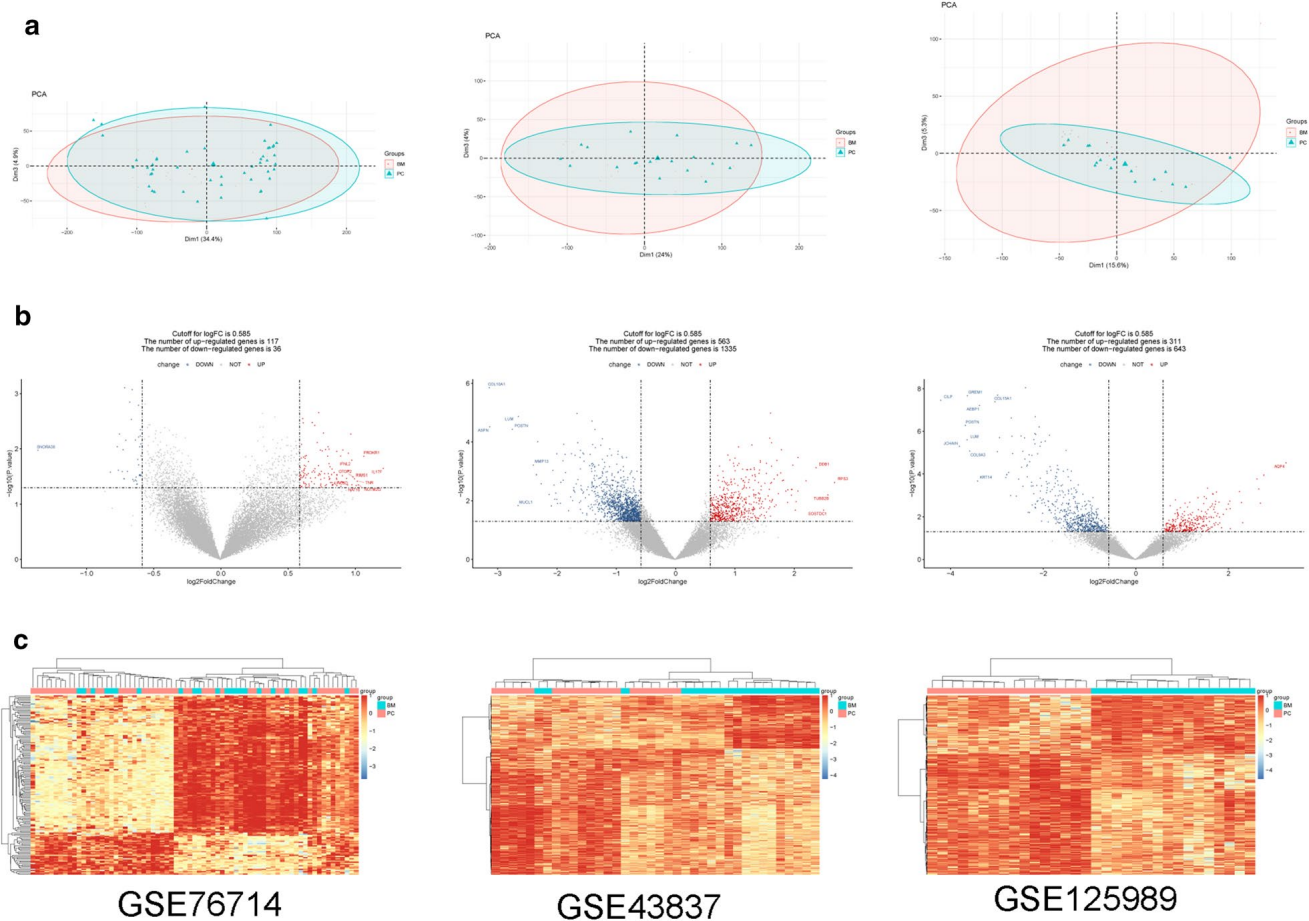
**a** Clustergrams of principal component analysis for three datasets. **b** The volcano plots for differentially expressed genes (DEGs) in three datasets. The red square represents upregulated DEGs, the blue circle represents downregulated DEGs, and the black triangle represents non-DEGs. The transverse dashed line is the p value, and the longitudinal dashed line is the fold change. The genes with the top tenfold changes are shown in the figure. **c** The heatmap of DEGs in three datasets. Red represents high expression and blue represents low expression

After preprocessing, the overall expression pattern of the samples was similar for each dataset. There was no significant batch effect among the samples in each dataset (Fig. 2a).

A total of 153 DEGs were obtained from the GSE76714 dataset, including 117 upregulated and 36 downregulated genes. Total 1898 DEGs were obtained from the GSE43837, including 563 upregulated and 1335 downregulated genes. Additionally, 954 DEGs were obtained from the GSE125989, including 311 upregulated genes and 643 downregulated genes. The volcano plots for DEGs are shown in Fig. 2b. The bidirectional hierarchical clustering heatmaps of DEGs is shown in Fig. 2c.

### Tumor immune microenvironment analysis

**Fig. 3:**
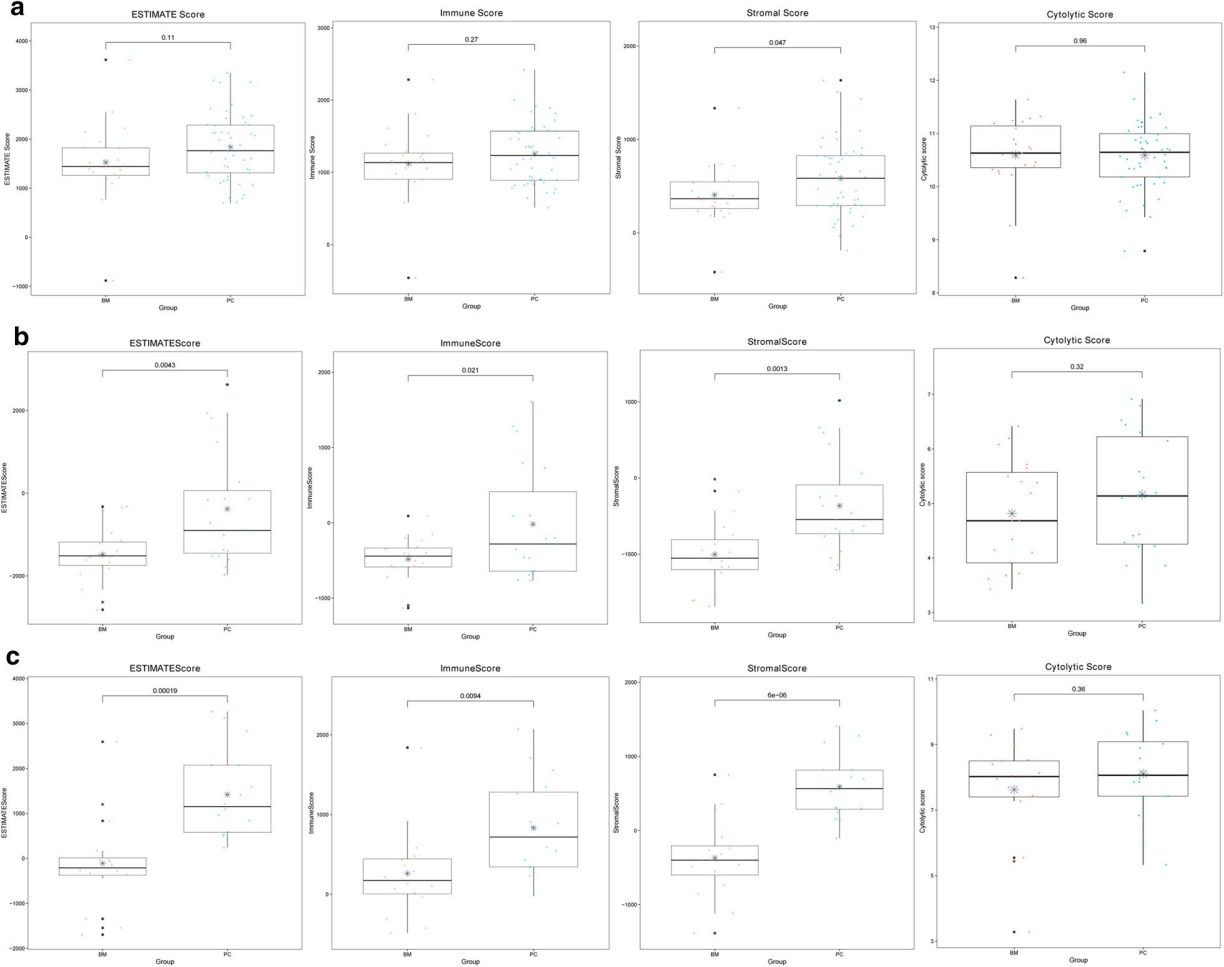
Box plots for stromal score, immune score, ESTIMATE score, and cytolytic activity score between brain metastases (BM) and primary cancer (PC) groups in GSE76714 (**a**), GSE43837 (**b**), and GSE125989 (**c**)

In GSE76714, the stromal score of brain metastases group was significantly lower than that of primary cancer group, while the immune score, ESTIMATE score, and cytolytic activity score showed no significant difference between two groups (Fig. 3a). For the datasets of GSE43837 and GSE125989, the stromal score, immune score, and ESTIMATE score in brain metastases group were significantly lower than that in primary cancer group. Additionally, cytolytic activity scores for the two groups were not significantly different (Fig. 3b, c). The result may indicate the difference of immune microenvironment between two groups.

### Immune cell infiltration abundance analysis

**Fig. 4:**
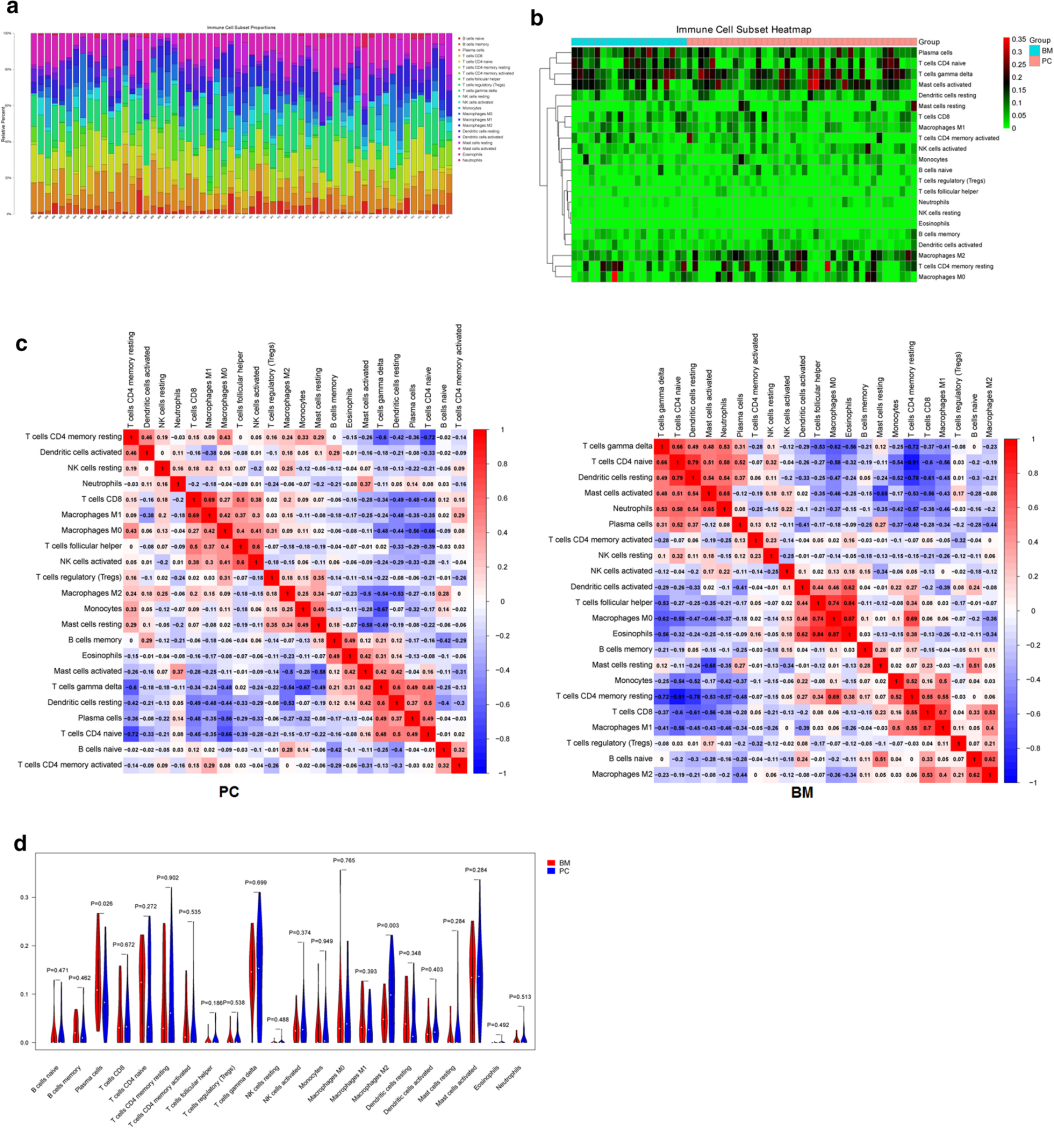
The barplot (**a**), clustering heatmap (**b**), correlation heatmap (**c**), and violin plot (**d**) of immune cell infiltration in GSE76714

The infiltration abundance matrix of 22 kinds of immune cells in all samples of GSE76714 was estimated using Cibersort algorithm. The result showed that among the 71 samples, 60 were valid, including 40 cases of PC and 20 cases of BM. The immune cell infiltration abundance for GSE76714 is shown in Fig. 4. The barplot (Fig. 4a) and clustering heatmap (Fig. 4b) showed that the infiltration rates of T cells gamma delta (green), T cells CD4 naive (yellow) and mast cells activated cells (pink) in each sample were relatively high. The correlation heatmap indicated that there existed difference in immune cell infiltration pattern between PC and BM groups (Fig. 4c). For instance, the correlation between NK cell activated and T cell helper in BM was very low (r = 0.02), while it was relatively higher in PC (r = 0.6). The violin plot showed that the infiltration ratio of plasma cells in brain metastases samples was significantly higher than that in primary cancer samples (p = 0.026), while the infiltration ratio of macrophages M2 cells in brain metastases samples was lower than that in primary cancer samples (p = 0.003) (Fig. 4d). For GSE43837 and GSE125989, there was not enough CIBERSORT for analysis.

### Identification of immune-related genes

**Fig. 5:**
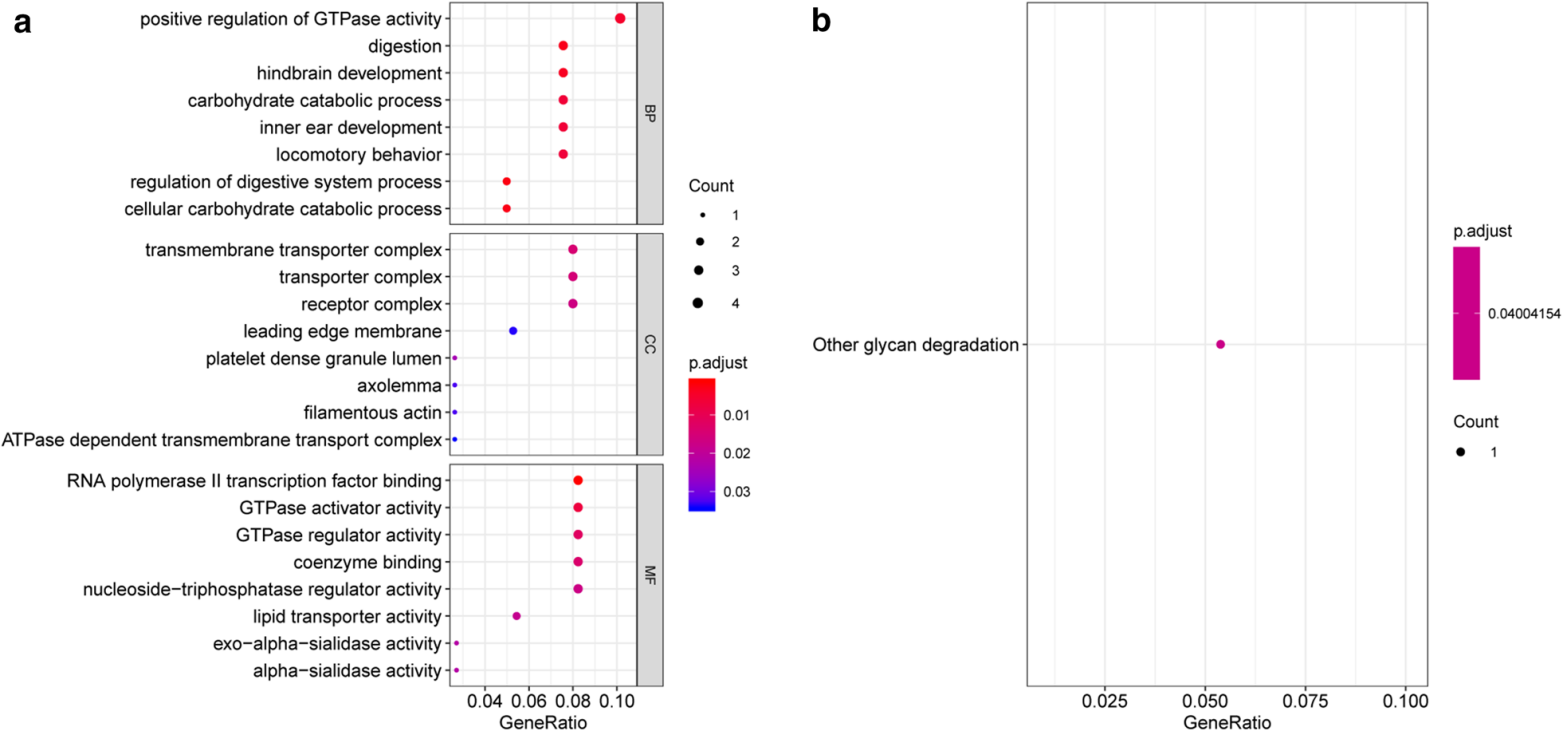
The GO function (**a**) and KEGG pathway (**b**) enriched by the immune-related genes

Correlation analysis was conducted between macrophages M2 cell infiltration and the expression of DEGs, and 42 immune-related genes were screened. T-test revealed that 27 genes, such as *THY1*, present significant differences in expression levels between two groups. These genes were significantly enriched in BP term associated with positive regulation of GTPase activity (*ALDH1A1*, *APC2*, *DOCK2* and *THY1*). Additionally, other glycan degradation pathway was significantly enriched (Fig. 5).

### TF-miRNA-mRNA network analysis

**Fig. 6:**
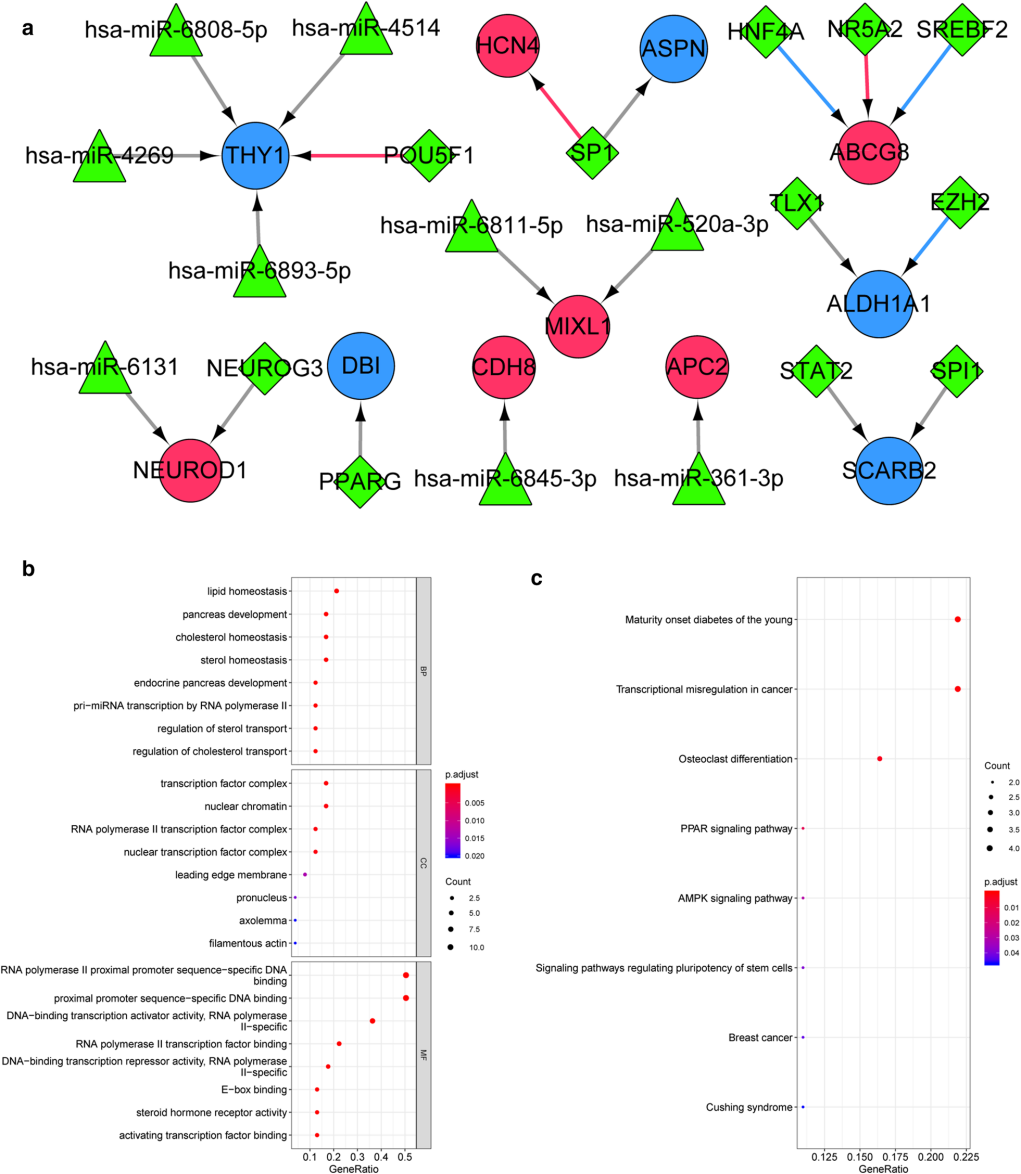
**a** The constructed transcription factor (TF)-miRNA-mRNA regulatory network. The red round nodes represent upregulated differentially expressed genes (DEGs); the blue round nodes represent downregulated DEGs; the green triangle nodes represent miRNAs; green rhombus nodes represent TF. The gray lines represent the interaction relationship; the red lines represent the activation relationship of TF-mRNA; the blue lines represent the inhibition relationship of TF-mRNA. B and C: The GO function (**b**) and KEGG pathway (**c**) enriched by the TF-mRNA

A total of 9 miRNA-mRNA pairs were predicted, which included 5 mRNAs and 9 miRNAs, such as miR-520a and miR-361-3p. In addition, 12 TF-mRNA pairs were obtained, involving 8 mRNAs and 11 TFs. Based on the miRNA-mRNA and TF-mRNA intraction pairs, a TF-miRNA-mRNA regulatory network was constructed (Fig. 6a). Function analysis showed that the TFs and mRNAs in the network were significantly enriched in lipid homeostasis, and cholesterol homeostasis associated BP terms (Fig. 6b). Moreover, they were involved in 8 pathways, such as transcriptional misregulation in cancer, PPAR signaling pathway, AMPK signaling pathway, and breast cancer (Fig. 6c).

### PPI network analysis

**Fig. 7:**
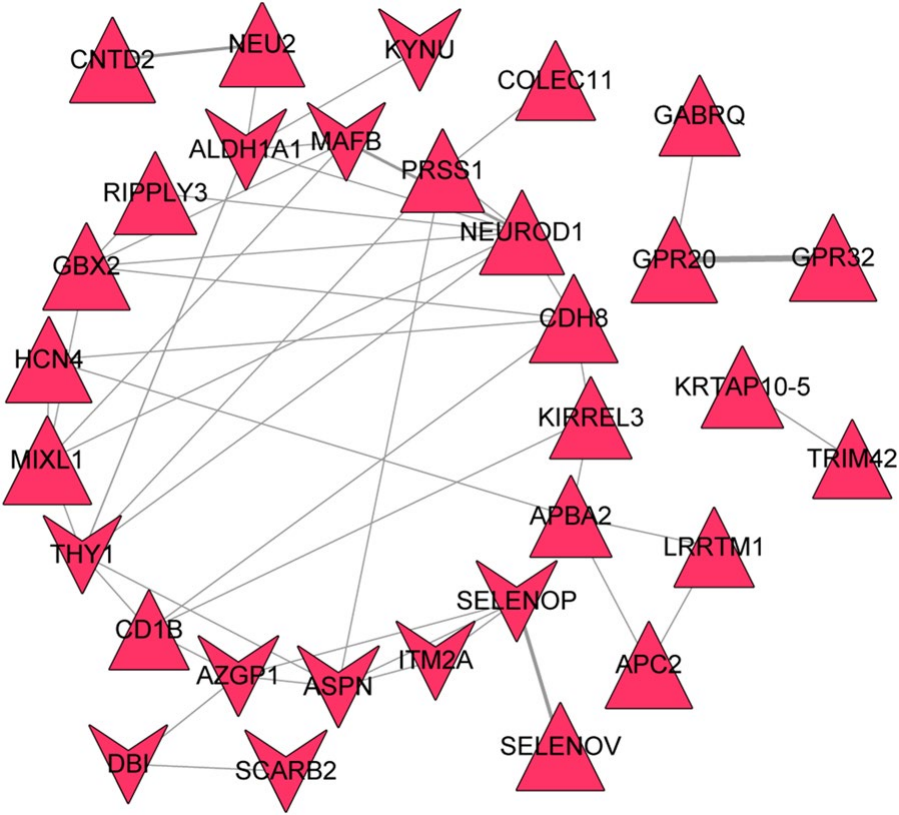
**a** The constructed protein–protein interaction (PPI) network of differentially expressed genes (DEGs) associated with macrophages M2. The triangle nodes represent upregulated DEGs; the red node represents DEGs associated with macrophages M2. **b** The thickness of the line is positively correlated to the relationship score obtained by STRING

Based on the immune-related genes, 46 PPI pairs were obtained and the constructed PPI network included 31 nodes (21 up-regulated and 10 down-regulated ones) (Fig. 7). Among the 31 nodes, *NEUROD1*, *THY1*, *ALDH1A1*, *GBX2*, *MIXL1*, *CDH8* and *ASPN* had degrees more than 5, and were considered as hub nodes.

### Drug-gene interaction analysis

**Fig. 8:**
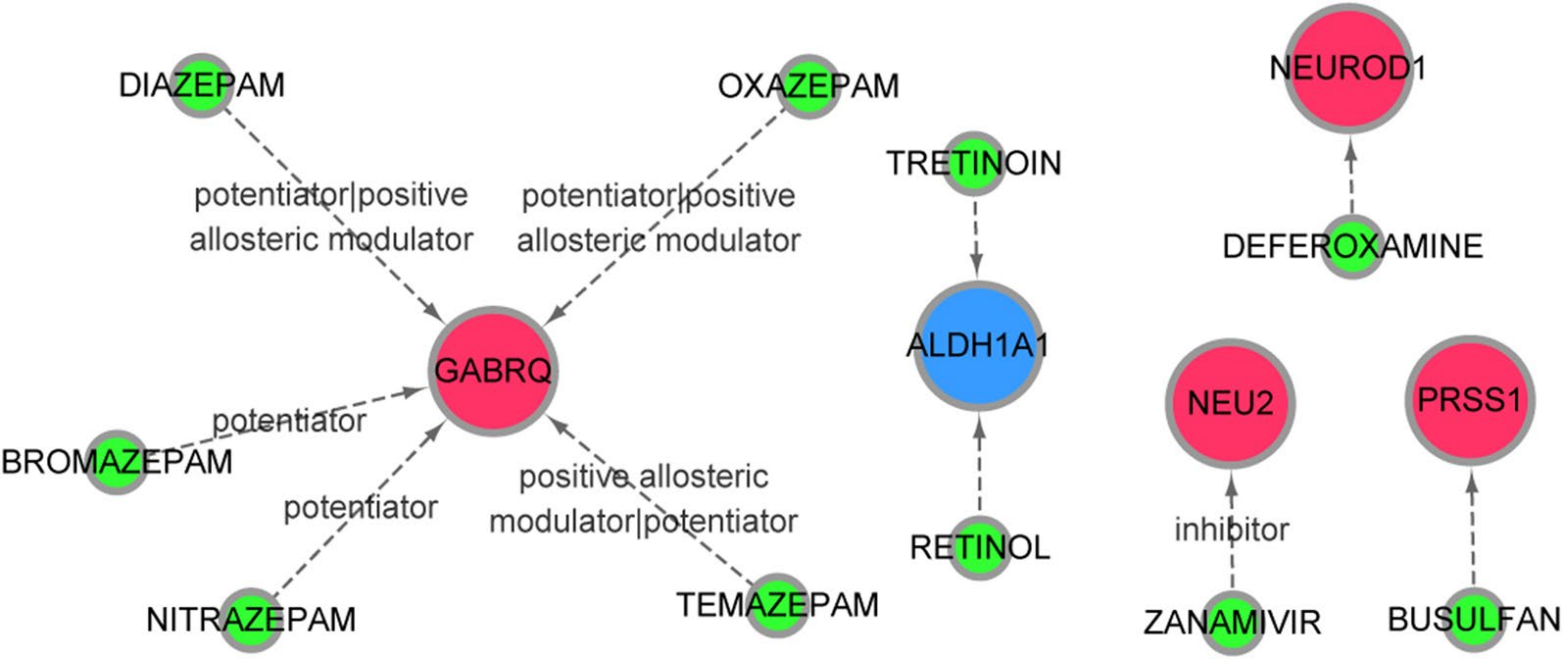
The constructed drug-gene interaction network. The red nodes represent the upregulated differentially expressed genes (DEGs) in macrophages M2. Blue nodes represent downregulated DEGs in macrophages M2. The green nodes represent small molecule drugs

A total of 10 drug-gene interaction pairs were identified based on the immune-related genes, which involved 10 drugs (busulfan, retinol, tretinoin, zanamivir, deferoxamine, temazepam, diazepam, oxazepam, bromazepam and nitrazepam) and 5 mRNAs (*PRSS1*, *ALDH1A1*, *NEU2*, *NEUROD1* and *GABRQ*). In detail, *GABRQ* interacted with temazepam, diazepam, oxazepam, bromazepam and nitrazepam; *ALDH1A1* interacted with tretinoin and retinol; *NEU2* interacted with zanamivir; *PRSS1* interacted with busulfan (Fig. 8).

### Metastatic recurrence survival analysis of key genes

**Fig. 9:**
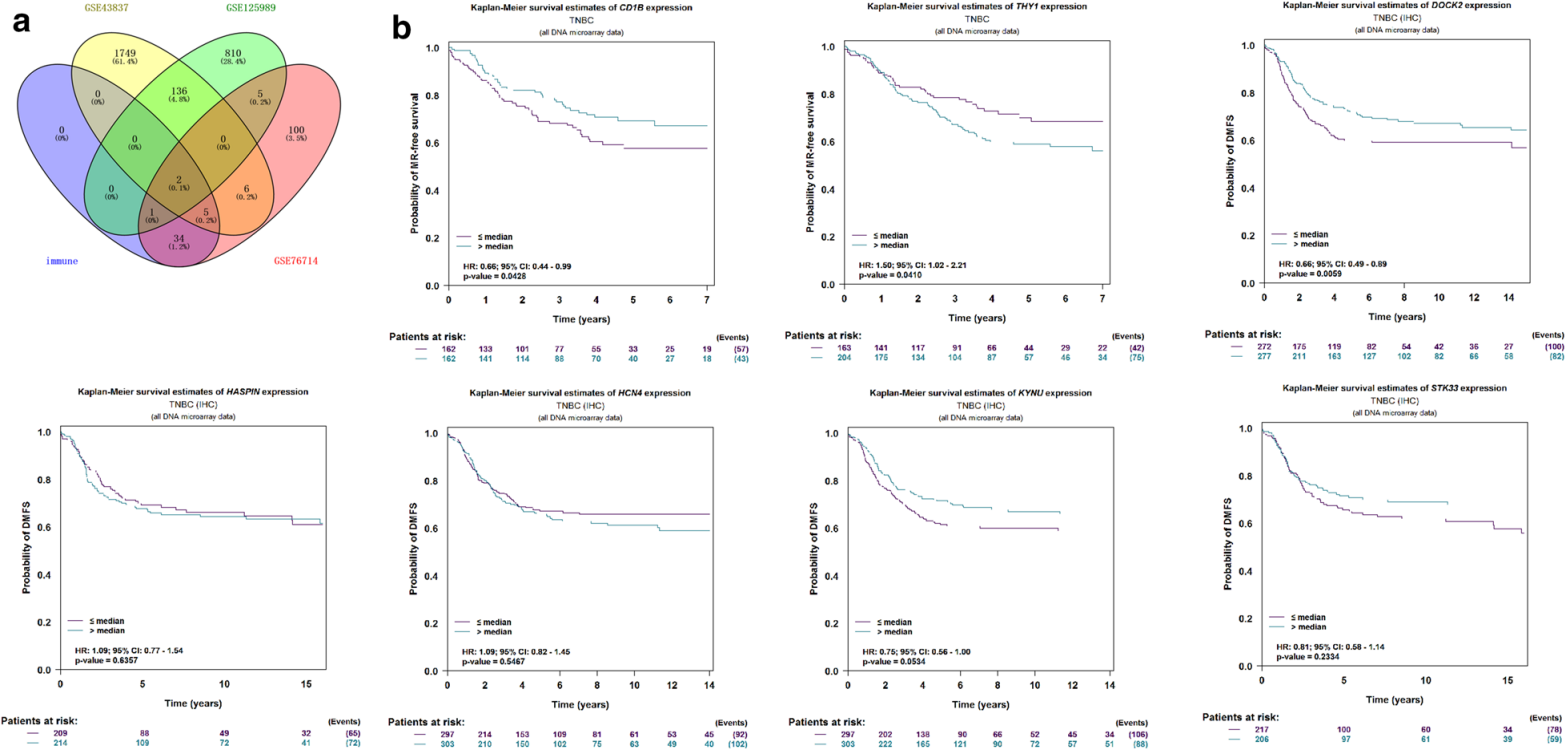
**a** Venn diagram of differentially expressed genes (DEGs) and DEGs in macrophages M2. **b** Metastatic recurrence survival curve for *CD1B*, *THY1*, *DOCK2*, *HCN4*, *HASPIN*, *STK33* and *KYNU*

The Venn diagram of the intersection of DEGs in the three datasets and immune-related genes is shown in Fig. 9a. The genes verified by GSE43817 dataset were *DOCK2*, *HCN4*, *HASPIN*, *STK33* and *KYNU*. The gene verified by GSE125989 dataset was *THY1*. The genes verified by the two datasets were *ASPN* and *CD1B*. These genes were considered as key genes and were performed metastatic recurrence survival analysis. Based on the analysis of the bc-GenExMiner v4.4 database, *CD1B*, *THY1* and *DOCK2* were found to affect the metastatic recurrence of triple-negative breast cancer. As shown in Fig. 9b, high expression of *THY1* was more likely to cause metastasis of breast cancer, while low expression of *CD1B* and *DOCK2* was likely to cause metastasis of breast cancer.

### Literature retrieval of key genes

Among the key genes, *ASPN*, *DOCK2*, *THY1* and *KYNU* were found to be associated with breast cancer based on NCBI Entrez database. Based on the GenCLiP 2.0 database, only *THY1* was associated with breast cancer.

## Discussion

Brain metastases commonly originate from breast, lung, and melanoma. However, brain metastases are hard to treat because most drugs cannot penetrate the blood brain barrier and often affect multiple areas of the brain [40]. Therefore, identification of new biomarkers may contribute to the development of individualized treatment. The present study is the first time to explore the immune-related genes in breast cancer with brain metastases. The present results showed that the tumor immune microenvironment in brain metastases was immunosuppressed compared with primary caner. Compared with primary cancer samples, the infiltration ratio of plasma cells in brain metastases samples was significantly higher, while the infiltration ratio of macrophages M2 cells in brain metastases samples was significantly lower. Total 42 immune-related genes were identified, such as *THY1* (Thy-1 cell surface antigen) and *NEU2* (neuraminidase 2). *THY1* was a hub protein in the PPI network. *NEU2* interacted with zanamivir. *CD1B* (CD1b molecule), *THY1* and *DOCK2 (dedicator of cytokinesis 2)* were found to be implicated in the metastatic recurrence of breast cancer.

The brain has long been regarded as an immune privileged organ, whereas, this view was revised since a lymphatic vessel network of brain tissues was found in the dura mater in mice [41, 42]. Our study suggested an immune suppressive environment in the brain metastases, partly exemplified by significantly decreased stromal score, immune score, and ESTIMATE score in brain metastases samples compared with that in primary cancer group. Our results were in accordance with a recent study of Kudo et al. [43], who coupled immune gene expression profiling and topological gene–gene network analysis and demonstrated an immune suppressive microenvironment in the brain metastases of non-small-cell lung cancer.

Tumor microenvironment is composed of various nonmalignant stromal cells, among which tumor-associated macrophages are the most prominent type of migratory hematopoietic cells [44]. In breast, the tumor-associated macrophages are primarily pro-tumorigenic M2-like macrophages, which promote the progression and metastasis of breast cancer by releasing various cytokines [45]. Plasma cells can affect antitumor immunity by regulating T-cell responses, or excluding immune-suppressive cell types to provide a permissive tumor microenvironment for CD8^+^ tumor-infiltrating lymphocytes, the key mediators of antitumor immunity [46]. Our results may devise new strategies for immunotherapy of breast cancer with brain metastases—by targeting macrophages or plasma cells.

In this study, 42 immune-related genes were identified, such as *THY1*, *NEU2*, *CD1B* and *DOCK2*. *THY1* was one of hub proteins in the PPI network. In PPI network, the topological placement of a protein is connected with its biological essentiality. The densely connected hub proteins are more likely to be essential proteins, which is referred to as the “centrality-lethality rule” [47]. THY1 is a glycophosphatidylinositol-anchored protein, which has been proposed to play important roles in cancers [48]. Function analysis showed that *THY1* was significantly enriched in function associated with positive regulation of GTPase activity. GTPases can be activated when binding to GTP. Once activated, GTPases carry out many functions in cells, such as the regulation of cell proliferation, apoptosis, and differentiation [49]. It has been reported that in the case of tumor progression, mutations in Ras related small GTPases can increase the proliferation, survival, and adhesion of tumor cells, tending toward a metastatic phenotype [50]. Additionally, high expression of *THY1* was more likely to cause metastasis of breast cancer. Taken together, we speculated that *THY1* may play a role in brain metastases of breast cancer via positive regulation of GTPase activity. Additionally, it mays sever as a prognostic indicator to predict the metastasis of breast cancer.

*NEU2* was involved in the pathway of other glycan degradation. In mammalian tissues, glycans exist in free forms or conjugated forms, which participate in various biological processes, such as host–pathogen interactions, cell migration and metastasis, and initiation of immune response [51]. Study has reported that glycan changes in malignant cells take many forms and mediate key pathophysiological events during various stages of tumor progression [52]. In the tumor environment, glycosylation changes allow tumor cells to usurp many development events, allowing tumor cells to invade and spread throughout the organism. Thus, we speculated that the upregulation of *NEU2* may be involved in brain metastases of breast cancer through pathway of other glycan degradation. Drug-gene interaction analysis showed that *NEU2* interacted with zanamivir. Zanamivir is an inhibitor of the enzyme neuraminidase, a surface glycoprotein necessary for the replication of type A and B influenza viruses [53]. Its role in cancer is rarely reported. Now we speculated that zanamivir may serve as an antineoplastic drug in breast cancer with brain metastases by targeting *NEU2.*

CD1B belongs to the group 1 CD1 family of transmembrane glycoproteins, and is associated with major histocompatibility complex class I-like molecules. CD1 molecules regulate the expression of some self- and foreign-lipid antigens to T-cell receptors on T cells [54]. A recent study has indicated that there are different expression patterns of CD1 molecules between tumor cells and normal cells [55]. More recently, low expression of *CD1B* was reported to be correlated with poorer biochemical recurrence-free survival in prostate cancer. Similar result was found in our study, that was, low expression of *CD1B* was likely to cause metastatic recurrence of breast cancer.

*DOCK2* is a member of the CDM protein family, which can regulate cell motility and cytokine production by activating Rac in mammalian hematopoietic cells. Additionally, *DOCK2* plays a critical role in the modulation of the immune system [56]. Hu et al. [57] have reported that low expression of *DOCK2* is associated with poorer prognosis of acute myeloid leukemia. Recent study reported that *DOCK2* hypermethylation was associated with biochemical recurrence after radical prostatectomy in prostate cancer [58]. To our knowledge, there was no study about the role of *DOCK2* in breast cancer. Together with our results, we speculated that *DOCK2* may be a prognostic marker of metastatic recurrence in breast cancer.

Despite these findings, there existed a limitation in this study. Due to lack of adequate clinical samples, there was no experimental evidence to support our analysis results. Thus, further experimental studies are needed to confirm our results.

## Conclusions

Our study indicated that tumor immune microenvironment in brain metastases of breast cancer was immunosuppressed compared with primary caner. Targeting macrophages or plasma cells may be new strategies for immunotherapy of breast cancer with brain metastases. *THY1* and *NEU2* may be potential therapeutic targets for breast cancer with brain metastases, and *THY1*, *CD1B* and *DOCK2* may serve as potential prognostic markers for improvement of brain metastases survival.

## Data Availability

The data that support the findings of this study are available from University of California Santa Cruz Genome Browser and GEO database.

## Abbreviations

GEO: gene expression omnibus
FC: fold change
barplot: relevant landscape map
pheatmap: clustering heatmap
corHeatmap: correlation heatmap
vioplot: violin plot
GO: gene ontology
BP: biological process
MF: molecular function
TF: transcription factor
CTD: comparative toxicogenomics database
PPI: protein–protein interaction
